# *Toxoplasma gondii *infection in workers occupationally exposed to unwashed raw fruits and vegetables: a case control seroprevalence study

**DOI:** 10.1186/1756-3305-4-235

**Published:** 2011-12-16

**Authors:** Cosme Alvarado-Esquivel, Sergio Estrada-Martínez, Oliver Liesenfeld

**Affiliations:** 1 Faculty of Medicine and Nutrition, Juárez University of Durango State. Avenida Universidad S/N. 34000 Durango, Dgo, Mexico; 2 Institute for Scientific Research, Juárez University of Durango State. Avenida Universidad S/N. 34000 Durango, Durango. Mexico; 3 Institute for Microbiology and Hygiene, Campus Benjamin Franklin, Charité Medical School, Hindenburgdamm 27. D-12203 Berlin, Germany. Present address: Roche Molecular Diagnostics, Pleasanton, CA. USA

**Keywords:** Case-control study, fruits workers, occupational exposure, seroprevalence, epidemiology

## Abstract

**Background:**

Through a case control seroprevalence study, we sought to determine the association of *Toxoplasma gondii *infection with occupational exposure to unwashed raw fruits and vegetables.

**Methods:**

Subjects, numbering 200, who worked growing or selling fruits and vegetables, and 400 control subjects matched by age, gender, and residence were examined by enzyme immunoassays for the presence of anti-*Toxoplasma *IgG and IgM antibodies. Socio-demographic, clinical, and behavioral characteristics from the study subjects were obtained.

**Results:**

Of the 200 fruit and vegetable workers, 15 (7.5%) of whom, and 31 (7.8%) of the 400 controls were positive for anti-*Toxoplasma *IgG antibodies (*P *= 0.96). Anti-*Toxoplasma *IgM antibodies were found in 2 (1%) of the fruit workers and in 11 (2.8%) of the control subjects (*P *= 0.23). Seroprevalence of *Toxoplasma *antibodies increased with age (*P *= 0.0004). In addition, seropositivity to *Toxoplasma *was associated with ill status (*P *= 0.04), chronic tonsillitis (*P *= 0.03), and reflex impairment (*P *= 0.03). Multivariate analysis showed that *Toxoplasma *infection was associated with consumption of raw meat (OR = 5.77; 95% CI: 1.15-28.79; *P *= 0.03), unwashed raw fruits (OR = 2.50; 95% CI: 1.11-5.63; *P *= 0.02), and living in a house with soil floors (OR = 3.10; 95% CI: 1.22-7.88; *P *= 0.01), whereas *Toxoplasma *infection was negatively associated with traveling abroad (OR = 0.28; 95% CI: 0.12-0.67; *P *= 0.005).

**Conclusions:**

This is the first report of seroprevalence and contributing factors for *Toxoplasma *infection in workers occupationally exposed to unwashed raw fruits and vegetables, and the results may help in the design of optimal preventive measures against *Toxoplasma *infection especially in female workers at reproductive age.

## Background

*Toxoplasma gondii *(*T. gondii*) infects humans worldwide [1-3]. Infections with *T. gondii *may result in asymptomatic latent infections or lymph node, ocular, or central nervous system disease [1-3]. We have been studying the epidemiology of *T. gondii *infection in humans [4-9] and other animals [10,11] in Durango, Mexico. Occupational exposure to parasite tissue cysts [12,13] and oocysts [13,14] in some population groups has been evaluated. Infections with *T. gondii *have been associated with the consumption of unwashed raw fruits and vegetables contaminated with oocysts in several countries [15-20]. Therefore, occupational exposure to unwashed raw fruits and vegetables could represent a hazard for *T. gondii *infection. To our knowledge, an association between occupational exposure to unwashed raw fruits and vegetables and *T. gondii *infection has not been evaluated. Therefore, through a case control exploratory study we sought to determine (1) the seroprevalence and levels of anti-*T. gondii *IgG antibodies in workers occupationally exposed to unwashed raw fruits and vegetables in Durango, Mexico, (2) the association of seropositivity to *T. gondii *with occupational exposure to unwashed raw fruits and vegetables, and (3) socio-demographic, clinical, and behavioral characteristics associated with *T. gondii *seropositivity in workers occupationally exposed to unwashed raw fruits and vegetables.

## Methods

### Study design and study populations

Through a case-controlled seroprevalence study design, we assessed the association of *T. gondii *infection with occupational exposure to unwashed raw fruits and vegetables in workers occupationally exposed to unwashed raw fruits and vegetables and control subjects in Durango, Mexico from December 2009 to November 2011.

#### Workers occupationally exposed to unwashed raw fruits and vegetables

The subjects, 200 in number, who worked growing (n = 100) or selling (n = 100) fruits or vegetables were included in the study. Workers selling fruits and vegetables worked in 40 fruit and vegetable shops in the 3 largest fruit markets in Durango City. These 3 markets sell fruits and vegetables to wholesalers and retailers and are the main distributors of fruits and vegetables in Durango State. Inclusion criteria for workers occupationally exposed to fruits and vegetables were: 1) to have been currently working in fruit shops or growing fruits and vegetables for at least 6 months, 2) aged 15 years and older, 3) any gender; 4) any socioeconomic level, and 5) who accepted to participate in the study. Of the 200 workers occupationally exposed to unwashed raw fruits and vegetables, 155 (77.5%) were male and 45 (22.5%) were female. The mean age of the workers was 42.13 ± 18.84 years old (range, 15-86 years).

#### Control subjects

Control subjects, who numbered 400, matched with workers by age, gender, and residence were included in the study. The mean age in controls was 42.13 ± 18.83 (range: 15-88) and comparable with that in workers (*P *= 1.00). Control subjects were obtained from the general population of Durango, Mexico.

### Ethical aspects

The study was approved by the Ethical Committee of the Faculty of Medicine in Durango City. The purpose and procedures of the study were explained to all participants, and a written informed consent was obtained from all of them.

### Socio-demographic, clinical and behavioral data

We explored the characteristics of the participants with the aid of a standardized questionnaire. Socio-demographic data included age, gender, place of birth, place of residence, residence area (urban, suburban, rural), educational level, and socioeconomic status. Contributing and confounding risk factors of behavioral data from all participants were also obtained. These factors included animal contacts, contact with cat feces, travellling in Mexico and abroad, meat consumption (pork, beef, goat, sheep, boar, chicken, turkey, pigeon, rabbit, venison, squirrel, horse, opossum, or other), degree of meat cooking, consumption of unpasteurized milk, dried or cured meat (ham, sausages, salami, or chorizo), unwashed raw vegetables, fruits, or untreated water, contact with soil (gardening or agriculture), and type of floors at home. Questions regarding contributing and confounding risk factors of behavioral data from all participants refer to "in their entire life". Clinical data included current suffering from any disease, presence or history of lymphadenopathy, frequent presence of headaches; history of blood transfusion, transplantation or surgery; and memory, reflex, hearing, and visual impairments.

### Laboratory tests

Serum samples were obtained from the participants and kept frozen at -20°C until analyzed. Serum samples were assayed by both qualitative and quantitative methods for anti-*T. gondii *IgG antibodies with a commercially available enzyme immunoassay "*Toxoplasma *IgG" kit (International Immuno-Diagnostics, Foster City, California). Anti-*T. gondii *IgG antibody levels were expressed as International Units (IU) per ml, and a result equal to or greater than 8 IU per ml was considered positive. Sera positive for *T. gondii *IgG were further tested for anti-*T. gondii *IgM antibodies by a commercially available enzyme immunoassay "*Toxoplasma *IgM" kit (International Immuno-Diagnostics, Foster City, California). All tests were performed following the instructions of the manufacturer.

### Statistical analysis

Results were analyzed with the aid of the software Epi Info version 3.5.1 and SPSS 15.0 (SPSS Inc. Chicago, Illinois). Age among the groups was compared by the student's *t *test. For comparison of the frequencies among the groups, the Yates Corrected Test and when indicated the Fisher Exact Test, were used. Bivariate and multivariate analyses were used to evaluate the association between the characteristics of the subjects and *T. gondii *infection. Variables were included in the multivariate analysis if they had a *P *value equal to or less than 0.20 in the bivariate analysis. Odd ratio (OR) and 95% confidence interval (CI) were calculated by multivariate analysis using multiple, unconditional, logistic regression. A *P *value less than 0.05 was considered statistically significant.

## Results

Of the 200 fruit and vegetable workers, 15 (7.5%) of whom and 31 (7.8%) of the 400 controls were positive for anti-*T. gondii *IgG antibodies. No statistically significant difference (*P *= 0.96) in seroprevalence of anti-*T. gondii *IgG antibodies between the groups was found. Anti-*T. gondii *IgG antibody levels were obtained from 12 of 15 seropositive fruit workers and from 26 of 31 seropositive control subjects. Of the 12 seropositive fruit workers, 8 (66.7%) of whom and 18 (69.2%) of the 26 seropositive controls had high levels (> 150 IU/ml) of anti-*T. gondii *IgG antibodies with no statistically significant difference among the groups (*P *= 0.84). Anti-*T. gondii *IgM antibodies were found in 2 (1%) of the fruit workers and in 11 (2.8%) of the control subjects. No statistically significant difference (*P *= 0.23) in seroprevalence of anti-*T. gondii *IgM antibodies between the groups was found. Seroprevalence of *T. gondii *infection in workers growing fruits and vegetables was comparable (*P *= 0.59) with that found in workers of fruit shops (9% vs 6%, respectively). Seroprevalence of *T. gondii *infection was comparable among workers of the 3 fruit markets: 3 of 34 (8.8%), 5 of 60 (8.3%), and 1 of 6 (16.7%) (*P *= 0.79). Seropositive workers were found in 7 (17.5%) of the 40 fruit shops studied.

General socio-demographic characteristics of the workers occupationally exposed to unwashed raw fruits and vegetables and control subjects are shown in Table 1. Seroprevalence of *T. gondii *antibodies increased with age in all groups. Other socio-demographic characteristics including gender, birth place, residence, educational level, and socioeconomic status did not show an association with *T. gondii *seropositivity.

**Table 1:** Socio-demographic characteristics of the study populations and seroprevalence of *T. gondii *infection.

	Fruit and vegetable workers (n = 200)					Controls (n = 400)					All (n = 600)				
	
			Prevalence of					Prevalence of					Prevalence of		
			*T. gondii *infection		*P*			*T. gondii *infection		*P*			*T. gondii *infection		*P*
												
Characteristic	**No**.	*%*	**No**.	*%*	value	**No**.	*%*	**No**.	*%*	value	**No**.	*%*	**No**.	*%*	value
Age groups (years)															
30 or less	74	37.0	4	5.4	0.06	148	37	10	6.8	0.001	222	37.0	14	6.3	0.0004
31-50	60	30.0	2	3.3		120	30	3	2.5		180	30.0	5	2.8	
51-70	41	20.5	7	17.1		84	21	8	9.5		125	20.8	15	12	
> 70	25	12.5	2	8.0		48	12	10	20.8		73	12.2	12	16.4	
Gender															
Male	155	77.5	12	7.7	1.00	310	77.5	27	8.7	0.26	465	77.5	39	8.4	0.29
Female	45	22.5	3	6.7		90	22.5	4	4.4		135	22.5	7	5.2	
Birth place															
Durango State	165	90.2	10	6.1	0.33	351	88.4	25	7.1	0.32	516	89.0	35	6.8	0.22
Other Mexican states	18	9.8	2	11.1		45	11.3	6	13.3		63	10.9	8	12.7	
Abroad	-					1	0.3	0	0.0		1	0.2	0	0	
Residence place															
Durango State	198	99.0	15	7.6	1.00	391	98.5	30	7.7	0.56	589	98.7	45	7.6	0.77
Other Mexican states	2	1.0	0	0.0		5	1.3	1	20.0		7	1.2	1	14.3	
Abroad	-					1	0.3	0			1	0.2	0	0	
Residence area															
Urban	132	67.3	9	6.8	0.42	300	77.5	23	7.7	0.88	432	74.1	32	7.4	0.56
Suburban	9	4.6	0	0.0		24	6.2	2	8.3		33	5.7	2	6.1	
Rural	55	28.1	6	10.9		63	16.3	6	9.5		118	20.2	12	10.2	
Educational level															
No education	15	7.5	2	13.3	0.56	16	4	3	18.8	0.22	31	5.2	5	16.1	0.20
1-6 years	100	50.0	9	9.0		104	26.2	8	7.7		204	34.2	17	8.3	
7-12 years	66	33.0	3	4.5		141	35.5	13	9.2		207	34.7	16	7.7	
> 12 years	19	9.5	1	5.3		136	34.3	7	5.1		155	26.0	8	5.2	
Socio-economic level															
Low	83	44.1	6	7.2	1.00	124	31.7	10	8.1	1.00	207	35.8	16	7.7	0.89
Medium	105	55.9	8	7.6		267	68.3	21	7.9		372	64.2	29	7.8	

With respect to clinical characteristics (Table 2), the seroprevalence of *T. gondii *infection was significantly (*P *= 0.04) higher in ill (11.8%) than in healthy (5.3%) control subjects. Seropositive subjects suffered from various underlying diseases including diabetes, arterial hypertension, spinal disease, chronic tonsillitis, and other diseases. However, chronic tonsillitis was the only disease associated with *T. gondii *seropositivity. The seroprevalence of *T. gondii *infection was significantly (*P *= 0.03) higher in patients with chronic tonsillitis (2/3; 66.7%) than that (15/154; 9.7%) in patients with other diseases. In control subjects, the seroprevalence of *T. gondii *infection was significantly (*P *= 0.03) higher in those suffering from reflex impairment (15.9%) than those without this clinical characteristic (6.2%). In the entire population (cases and controls), the seroprevalence of *T. gondii *infection was also significantly (*P *= 0.03) higher in subjects suffering from reflex impairment (14.9%) than those without this clinical feature (6.8%). Other clinical characteristics including presence or history of lymphadenopathy, frequent presence of headaches; history of blood transfusion, transplantation or surgery; and memory, hearing, and visual impairments did not show an association with *T. gondii *seropositivity.

**Table 2:** Bivariate analysis of clinical data and infection with *T. gondii *in fruit and vegetable workers and controls.

	Workers (n = 200)				Controls (n = 400)				All (n = 600)			
	
	No. of	Prevalence of			No. of	Prevalence of			No. of	Prevalence of		
	subjects	*T. gondii *infection		*P*	subjects	*T. gondii *infection		*P*	subjects	*T. gondii *infection		*P*
Characteristic	tested	**No**.	%	value	tested	**No**.	%	value	tested	**No**.	%	value
Health status												
Healthy	113	11	9.7	0.53	266	14	5.3	0.04	379	25	6.6	0.13
Ill	47	4	8.5		110	13	11.8		157	17	10.8	
Lymphadenopathy ever												
Yes	30	3	10.0	0.46	68	4	5.9	0.44	98	7	7.1	0.93
No	115	9	7.8		296	22	7.4		411	31	7.5	
Headaches frequently												
Yes	37	5	13.5	0.15	104	7	6.7	0.93	141	12	8.5	0.77
No	109	7	6.4		262	20	7.6		371	27	7.3	
Blood transfusion												
Yes	23	3	13.0	0.24	33	1	3.0	0.49	56	4	7.1	1.00
No	176	12	6.8		365	30	8.2		541	42	7.8	
Transplantation												
Yes	0	0	0.0	-	1	0	0.0	1.00	1	0	0	1.00
No	194	15	7.7		397	31	7.8		591	46	7.8	
Surgery ever												
Yes	60	7	11.7	0.63	165	15	9.1	0.35	225	22	9.8	0.25
No	99	8	8.1		200	12	6.0		299	20	6.7	
Memory impairment												
Yes	49	7	14.3	0.11	75	6	8.0	0.99	124	13	10.5	0.30
No	115	8	7.0		306	22	7.2		421	30	7.1	
Reflex impairment												
Yes	30	4	13.3	0.28	44	7	15.9	0.03	74	11	14.9	0.03
No	134	11	8.2		337	21	6.2		471	32	6.8	
Hearing impairment												
Yes	31	3	9.7	0.54	63	6	9.5	0.43	94	9	9.6	0.61
No	127	11	8.7		320	22	6.9		447	33	7.4	
Visual impairment												
Yes	66	5	7.6	0.98	164	17	10.4	0.07	230	22	9.6	0.18
No	91	8	8.8		219	11	5.0		310	19	6.1	

Bivariate analysis showed a number of behavioral characteristics with a *P *value equal to or less than 0.20 including cats at home, soil floors at home, traveling abroad, consumption of boar, pigeon, deer, and squirrel meats, consumption of raw meat and unwashed raw fruits, and consumption of ham and salami. Results of multivariate analysis of selected behavioral characteristics are shown in Table 3. Seropositivity to *T. gondii *was associated with consumption of raw meat (OR = 5.77; 95% CI: 1.15-28.79; *P *= 0.03) and unwashed raw fruits (OR = 2.50; 95% CI: 1.11-5.63; *P *= 0.02), and living in a house with soil floors (OR = 3.10; 95% CI: 1.22-7.88; *P *= 0.01), whereas traveling abroad was negatively associated with *T. gondii *seropositivity (OR = 0.28; 95% CI: 0.12-0.67; *P *= 0.005). Other behavioral characteristics including consumption of any type of meat; unpasteurized milk, untreated water, unwashed raw vegetables; or contact with cats or other animals did not show any association with *T. gondii *seropositivity.

**Table 3:** Multivariate analysis of selected characteristics of the participants and their association with *T. gondii *infection.

	Adjusted	95%	
	odds	Confidence	*P*
Characteristic^a^	ratio^b^	interval	value
Cats at home	1.62	0.78-3.39	0.19
Travel abroad	0.28	0.12-0.67	0.005
Boar meat consumption	1.41	0.46-4.26	0.53
Pigeon meat consumption	1.48	0.56-3.91	0.41
Venison consumption	0.86	0.36-2.07	0.75
Squirrel meat consumption	1.27	0.49-3.25	0.61
Raw meat consumption	5.77	1.15-28.79	0.03
Ham consumption	0.60	0.17-2.13	0.43
Salami consumption	0.64	0.29-1.43	0.28
Unwashed raw fruits	2.50	1.11-5.63	0.02
Soil floor at home	3.10	1.22-7.88	0.01

^a^The variables included were those with a p < 0.20 obtained in the bivariate analysis.

^b^Adjusted by age and the rest of characteristics included in this Table.

## Discussion

We found comparable seroprevalences and levels of anti-*T. gondii *IgG antibodies and seroprevalences of anti-*T. gondii *IgM antibodies among workers occupationally exposed to unwashed raw fruits and vegetables and their controls. Although unwashed raw fruits and vegetables could be contaminated with *T. gondii*, our results suggest that handling of these products seems not to be a major occupational risk for *T. gondii *infection. The lack of association of *T. gondii *seropositivity with exposure to fruits and vegetables could probably be explained by both a low frequency and a low concentration of oocysts in the fruits and vegetables handled. In addition, there is no direct entry of *T. gondii *into the body by handling fruits or vegetables as occurs by eating these products. Presence of oocysts in fruits may depend on contact of fruits with contaminated soil. Therefore, the low frequency of *T. gondii *seropositivity in our fruit workers might also be explained by the fact that workers collect fruits more frequently from trees than from the soil. Other factors might be involved in *T. gondii *transmission in fruit workers including frequency of eating fruits and vegetables, washing hands before eating, or other unknown factors. Further research to evaluate the association of *T. gondii *infection with occupational exposure to unwashed raw fruits and vegetables is needed.

Results of the present study indicate that some factors including consumption of raw meat and unwashed raw fruits, and living in a house with soil floors play a more important contributing role in *T. gondii *infection than handling unwashed raw fruits and vegetables. Multivariate analysis showed that seropositivity to *T. gondii *was associated with consumption of raw meat. Eating raw meat is a major cause of *T. gondii *infection [1,3]. Moreover, multivariate analysis showed that consumption of unwashed raw fruits was associated with *T. gondii *infection. Remarkably, we have repeatedly found an association of *T. gondii *infection with consumption of unwashed raw fruits in Durango, Mexico. In a previous study of psychiatric patients, we found a positive association of *T. gondii *infection with unwashed raw fruit consumption [4]. Similarly, in a recent study, an association between *T. gondii *infection and consumption of unwashed fruits was found in workers occupationally exposed to water, sewage, and soil [13]. Our results suggest that occupational exposure to raw fruits and vegetables per se does not seem to contribute substantially in increasing the seroprevalence of *T. gondii *infection, but seroprevalence increases substantially when eating unwashed raw fruits. It is known that *T. gondii *infection can be prevented in large part by peeling or thoroughly washing fruits and vegetables before eating [21]. Consumption of unwashed raw fruit has epidemiological concern in fruit workers especially in female fruit workers during the reproductive age.

Contaminated fruits and vegetables may represent a risk of infection in animals consumed by humans. Unwashed raw fruits and vegetables are frequently used to feed pigs in backyards in Durango. In fact, in a recent study, serological evidence of *T. gondii *infection in pigs in Durango was reported [11]. Furthermore, in the present work, an association between *T. gondii *infection and soil floors at home was found. In a previous study of pregnant women attending a public hospital in Durango City, we found that *T. gondii *infection was associated with living in a house with soil floors [5]. In a further study of pregnant women of 9 communities in rural Durango State, a similar association was found [7]. Therefore, our results confirm that living in a house with soil floors contributes to the increase in seroprevalence of *T. gondii *infection in Durango State. In contrast, our study showed that *T. gondii *infection was negatively associated with travelling abroad. This finding suggests that most *T. gondii *infections may have been acquired in Mexico.

The seroprevalence of *T. gondii *infection increased significantly with age in the subjects studied. This finding follows a typical tendency of infection in humans as reported elsewhere [3,9]. In general, the seroprevalence of *T. gondii *infection was higher in ill than in healthy subjects. In particular, the seroprevalence of *T. gondii *infection was higher in patients with chronic tonsillitis than in patients with other diseases. There is scarce and conflicting information in the role of *T. gondii *in the etiology of tonsillitis [22,23]. In the present study, there were few cases of chronic tonsillitis; hence, further research with a larger sample size should be conducted to determine the association of *T. gondii *with chronic tonsillitis.

Remarkably, the seroprevalence of *T. gondii *infection was higher in subjects with reflex impairment than in those without this clinical feature. This finding agrees with those reported in 2 previous studies in Durango, Mexico. In a first study of patients with vision impairment, those with reflex impairment had a significantly higher frequency of *T. gondii *infection than those with normal reflexes [24]. In a second study of patients suffering from liver disease, the seroprevalence of *T. gondii *infection was higher in those with reflex impairment than in those without this impairment [8]. Several reports indicate that *T. gondii *infection may affect the reaction time in infected individuals. In a double blind study, Havlícek et al [25] reported significantly longer reaction times of subjects with latent toxoplasmosis in comparison with those of controls. Novotná et al [26] reported that heterozygous men with both the RhD plus and the RhD minus alleles were protected against prolongation of reaction times caused by infection with *T. gondii*. In a further study of men and women, Flegr et al [27] confirmed that RhD-positive subjects were less sensitive to the influence of latent toxoplasmosis on reaction times than RhD-negative subjects. In the present study, the associations of *T. gondii *seropositivity with several clinical characteristics including ill status, chronic tonsillitis, and reflex impairment suggest that *T. gondii *infection is impacting the health of our population.

## Conclusions

This is the first report of contributing factors for *T. gondii *infection in workers occupationally exposed to unwashed raw fruits and vegetables. The results may help in the design of optimal preventive measures.

## Competing interests

The authors declare that they have no competing interests.

## Authors' contributions

CAE conceived and designed the study protocol, participated in the coordination and management of the study, applied the questionnaires, performed the laboratory tests, the data analysis and statistical analysis, and wrote the manuscript. SEM performed the statistical analysis. OL performed the data analysis and wrote the manuscript. All authors read and approved the final version of the manuscript.
